# Supplementary material for: Dynalign II: common secondary structure prediction for RNA homologs with domain insertions
Source: Nucleic Acids Res. 2014 Nov 21;42(22):13939–48. doi: 10.1093/nar/gku1172 (PMC4267632; doi:10.1093/nar/gku1172)
Supplement: SUPPLEMENTARY DATA [file supp_gku1172_nar-02021-z-2014-File012.zip › manual/RNA_class/html/class_r_n_a.html]

RNAstructure Classes: RNA Class Reference

- Main Page
- Classes
- Files

- Class List
- Class Hierarchy
- Class Members

# RNA Class Reference

RNA Class.
More...

`#include <RNA.h>`

Inherits Thermodynamics.

Inherited by HybridRNA, and Oligowalk\_object.

List of all members.

|  |  |
| --- | --- |
|  |  |
| Public Member Functions | |
|  | RNA (const char sequence[], const bool IsRNA=true) |
|  | Constructor - user provides a sequence as a c string. |
|  | RNA (const char filename[], const int type, const bool IsRNA=true) |
|  | Constructor - user provides a filename for existing file as a c string. |
|  | RNA (const bool IsRNA=true) |
| int | GetErrorCode () |
|  | Return an error code, where a return of zero is no error. |
| char \* | GetErrorMessage (const int error) |
|  | Return error messages based on code from GetErrorCode and other error codes. |
| void | SetModifier (std::string modifier) |
| std::string | GetErrorMessageString (const int error) |
|  | Return error messages based on code from GetErrorCode and other error codes. |
| int | SpecifyPair (const int i, const int j, const int structurenumber=1) |
|  | Specify a base pair between nucleotides i and j. |
| int | RemovePairs (const int structurenumber=1) |
|  | Remove all the current base pairs in a specified structure. |
| int | RemoveBasePair (const int i, const int structurenumber=1) |
|  | Remove a specified pair in a specified structure. |
| double | CalculateFreeEnergy (const int structurenumber=1, const bool UseSimpleMBLoopRules=false) |
|  | Return the predicted Gibb's free energy change for structure # structurenumber, defaulted to 1. |
| int | WriteThermodynamicDetails (const char filename[], const bool UseSimpleMBLoopRules=false) |
|  | Calculate the folding free energy change for all structures and write the details of the calculation to a file. |
| int | FoldSingleStrand (const float percent, const int maximumstructures, const int window, const char savefile[]="", const int maxinternalloopsize=30, bool mfeonly=false) |
|  | Predict the lowest free energy secondary structure and generate suboptimal structures using a heuristic. |
| int | GenerateAllSuboptimalStructures (const float percent, const double deltaG) |
|  | Predict the lowest free energy secondary structure and generate all suboptimal structures. |
| int | MaximizeExpectedAccuracy (const double maxPercent, const int maxStructures, const int window, const double gamma=1.0) |
|  | Predict the structure with maximum expected accuracy and suboptimal structures. |
| int | PartitionFunction (const char savefile[]="", double temperature=-10.0) |
|  | Predict the partition function for a sequence. |
| int | PredictProbablePairs (const float probability=0) |
|  | Predict structures containing highly probable pairs. |
| int | ProbKnot (int iterations=1, int MinHelixLength=1) |
|  | Predict maximum expected accuracy structures that contain pseudoknots. |
| int | ReFoldSingleStrand (const float percent, const int maximumstructures, const int window) |
|  | Re-predict the lowest free energy secondary structure and generate suboptimal structures using a heuristic. |
| int | Stochastic (const int structures, const int seed=1) |
|  | Sample structures from the Boltzman ensemable. |
| int | ForceDoubleStranded (const int i) |
|  | Force a nucleotide to be double stranded (base paired). |
| int | ForceFMNCleavage (const int i) |
|  | Indicate a nucleotide that is accessible to FMN cleavage (a U in GU pair). |
| int | ForceMaximumPairingDistance (const int distance) |
|  | Force a maximum distance between apired nucleotides. |
| int | ForceModification (const int i) |
|  | Force modification for a nucleotide. |
| int | ForcePair (const int i, const int j) |
|  | Force a pair between two nucleotides. |
| int | ForceProhibitPair (const int i, const int j) |
|  | Prohibit a pair between two nucleotides. |
| int | ForceSingleStranded (const int i) |
|  | Force a nucleotide to be single stranded. |
| int | GetForcedDoubleStranded (const int constraintnumber) |
|  | Return a nucleotide that is forced double stranded. |
| int | GetForcedFMNCleavage (const int constraintnumber) |
|  | Return a nucleotide that is accessible to FMN cleavage. |
| int | GetForcedModification (const int constraintnumber) |
|  | Return a nucleotide that is accessible to modification. |
| int | GetForcedPair (const int constraintnumber, const bool fiveprime) |
|  | Return a nucleotide in a forced pair. |
| int | GetForcedProhibitedPair (const int constraintnumber, const bool fiveprime) |
|  | Return a nucleotide in a prohibited pair. |
| int | GetForcedSingleStranded (const int constraintnumber) |
|  | Return a nucleotide that is forced single stranded. |
| int | GetMaximumPairingDistance () |
|  | Return the maximum pairing distance. |
| int | GetNumberOfForcedDoubleStranded () |
|  | Return the number of nucletides forced to be paired. |
| int | GetNumberOfForcedFMNCleavages () |
|  | Return the number of nucleotides accessible to FMN cleavage. |
| int | GetNumberOfForcedModifications () |
|  | Return the number of nucleotides accessible to chemical modification. |
| int | GetNumberOfForcedPairs () |
|  | Return the number of forced base pairs. |
| int | GetNumberOfForcedProhibitedPairs () |
|  | Return the number of prohibited base pairs. |
| int | GetNumberOfForcedSingleStranded () |
|  | Return the number of nucleotides that are not allowed to pair. |
| int | ReadConstraints (const char filename[]) |
|  | Read a set of folding constraints to disk in a plain text file. |
| int | ReadSHAPE (const char filename[], const double parameter1, const double parameter2, std::string modifier="SHAPE", const bool IsPseudoEnergy=true) |
|  | Read SHAPE data from disk. |
| int | ReadSHAPE (const char filename[], const double parameter1, const double parameter2, const double ssm, const double ssb, std::string modifier="SHAPE") |
|  | Read SHAPE data from disk including single-stranded SHAPE pseudo free energys. |
| int | ReadDSO (const char filename[]) |
|  | Read double strand offset data from disk. |
| int | ReadSSO (const char filename[]) |
|  | Read single strand offset data from disk. |
| int | ReadExperimentalPairBonus (const char filename[], double const experimentalOffset, double const experimentalScaling) |
|  | Read experimental pair bonuses from disk. |
| void | RemoveConstraints () |
|  | Remove all folding constraints. |
| int | SetExtrinsic (int i, int j, double k) |
|  | Add extrinsic restraints for partition function calculations. |
| int | WriteConstraints (const char filename[]) |
|  | Write the current set of folding constraints to disk in a plain text file. |
| int | AddComment (const char comment[], const int structurenumber=1) |
|  | Add a comment associated with a structure. |
| int | WriteCt (const char filename[], bool append=false) |
|  | Write a ct file of the structures. |
| int | WriteDotBracket (const char filename[]) |
|  | Write dot-bracket file of structures. |
| int | BreakPseudoknot (const bool minimum\_energy=true, const int structurenumber=0) |
|  | Break any pseudoknots that might be in a structure. |
| bool | ContainsPseudoknot (const int structurenumber) |
|  | Report if there are any pseudoknots in a structure. |
| double | GetEnsembleEnergy () |
|  | Get the ensemble folding free energy change. |
| double | GetFreeEnergy (const int structurenumber) |
|  | Get the folding free energy change for a predicted structure. |
| int | GetPair (const int i, const int structurenumber=1) |
|  | Get the nucleotide to which the specified nucleotide is paired. |
| double | GetPairEnergy (const int i, const int j) |
|  | Get the lowest folding free energy possible for a structure containing pair i-j. |
| double | GetPairProbability (const int i, const int j) |
|  | Get a base pair probability. |
| int | GetStructureNumber () |
|  | Get the total number of specified or predicted structures. |
| int | DetermineDrawingCoordinates (const int height, const int width, const int structurenumber=1) |
|  | Determine the coordinates for drawing a secondary structure. |
| std::string | GetCommentString (const int structurenumber=1) |
|  | Provide the comment from the ct file as a string. |
| int | GetNucleotideXCoordinate (const int i) |
|  | Get the X coordinate for nucleotide i for drawing a structure. |
| int | GetNucleotideYCoordinate (const int i) |
|  | Get the Y coordinate for nucleotide i for drawing a structure. |
| int | GetLabelXCoordinate (const int i) |
|  | Get the X coordinate for placing the nucleotide index label specified by i. |
| int | GetLabelYCoordinate (const int i) |
|  | Get the Y coordinate for placing the nucleotide index label specified by i. |
| char | GetNucleotide (const int i) |
| int | GetSequenceLength () |
|  | Get the total length of the sequence. |
| bool | GetBackboneType () |
|  | Get the backbone type. |
| structure \* | GetStructure () |
| void | SetProgress (TProgressDialog &Progress) |
| void | StopProgress () |
| TProgressDialog \* | GetProgress () |
|  | ~RNA () |
|  | Destructor. |
| Protected Member Functions | |
| int | FileReader (const char filename[], const int type) |
| Protected Attributes | |
| int | ErrorCode |
| TProgressDialog \* | progress |
| Private Attributes | |
| structure \* | ct |
| bool | partitionfunctionallocated |
| bool | energyallocated |
| PFPRECISION \* | w5 |
| PFPRECISION \* | w3 |
| PFPRECISION \*\* | wca |
| pfdatatable \* | pfdata |
| pfunctionclass \* | w |
| pfunctionclass \* | v |
| pfunctionclass \* | wmb |
| pfunctionclass \* | wl |
| pfunctionclass \* | wmbl |
| pfunctionclass \* | wcoax |
| PFPRECISION | Q |
| arrayclass \* | ew2 |
| arrayclass \* | ewmb2 |
| integersize \* | ew5 |
| integersize \* | ew3 |
| int | vmin |
| arrayclass \* | ev |
| arrayclass \* | ew |
| arrayclass \* | ewmb |
| bool \* | lfce |
| bool \* | mod |
| forceclass \* | fce |
| coordinates \* | structurecoordinates |
| bool | drawallocated |

---

## Detailed Description

RNA Class.

The RNA class provides an entry point for all the single sequence operations of RNAstructure. 

---

## Constructor & Destructor Documentation

|  |  |  |  |  |
| --- | --- | --- | --- | --- |
| RNA::RNA | ( | const char | *sequence*[], |  |
|  |  | const bool | *IsRNA* = `true` |  |
|  | ) |  |  |  |

Constructor - user provides a sequence as a c string.

Input sequence should contain A,C,G,T,U,a,c,g,t,u,x,X. Capitalization makes no difference. T=t=u=U. If IsRNA is true, the backbone is RNA, so U is assumed. If IsRNA is false, the backbone is DNA, so T is assumed. x=X= nucleotide that neither stacks nor pairs. For now, any unknown nuc is considered 'X'. Note that sequences will subsequently be indexed starting at 1 (like a biologist), so that the 0th position in the sequence array will be nucleotide 1.

**Parameters:**
:   |  |  |  |
    | --- | --- | --- |
    |  | *sequence* | is a NULL terminated c string. |
    |  | *IsRNA* | is a bool that indicates whether this sequence is RNA or DNA. true=RNA. false=DNA. Default is true. |

|  |  |  |  |  |
| --- | --- | --- | --- | --- |
| RNA::RNA | ( | const char | *filename*[], |  |
|  |  | const int | *type*, |  |
|  |  | const bool | *IsRNA* = `true` |  |
|  | ) |  |  |  |

Constructor - user provides a filename for existing file as a c string.

The existing file, specified by filename, can either be a ct file, a sequence, or an RNAstructure save file. Therefore, the user provides a flag for the file: type = 1 => .ct file, type = 2 => .seq file, type = 3 => partition function save (.pfs) file, type = 4 => folding save file (.sav). This constructor generates internal error codes that can be accessed by GetErrorCode() after the constructor is called. 0 = no error. The errorcode can be resolved to a c string using GetErrorMessage. Note that the contructor needs to be explicitly told, via IsRNA, what the backbone is because files do not store this information. Note also that save files explicitly store the thermodynamic parameters, therefore changing the backbone type as compaared to the original calculation will not change structure predictions.

**Parameters:**
:   |  |  |  |
    | --- | --- | --- |
    |  | *filename* | is null terminated c string. |
    |  | *type* | is an integer that indicates the file type. |
    |  | *IsRNA* | is a bool that indicates whether this sequence is RNA or DNA. true=RNA. false=DNA. Default is true. |

|  |  |  |  |  |  |
| --- | --- | --- | --- | --- | --- |
| RNA::RNA | ( | const bool | *IsRNA* = `true` | ) |  |

Default Constructor - user provides nothing. This basic constructor is provided for bimolecular folding and should not generally need to be accessed by end users of the RNA class.

**Parameters:**
:   |  |  |  |
    | --- | --- | --- |
    |  | *IsRNA* | is a bool that indicates whether this sequence is RNA or DNA. true=RNA. false=DNA. Default is true. |

|  |  |  |  |  |
| --- | --- | --- | --- | --- |
| RNA::~RNA | ( |  | ) |  |

Destructor.

The destructor automatically cleans up all allocated memory for predicted or specified structures.

---

## Member Function Documentation

|  |  |  |  |  |
| --- | --- | --- | --- | --- |
| int RNA::AddComment | ( | const char | *comment*[], |  |
|  |  | const int | *structurenumber* = `1` |  |
|  | ) |  |  |  |

Add a comment associated with a structure.

This comment will appear in the .ct file. The comment is appended to any existing comments, like titles read from .seq files. This function is especially useful if the constructor is used in which a character array is provided with the sequence. In that case, there is no sequence title read. The function retuns 0 in the case of no errors, or 3 if the structurenumber is invalid. An error message can be retrieved using GetErrorMessage() called with the errorcode.

**Parameters:**
:   |  |  |  |
    | --- | --- | --- |
    |  | *comment* | is a character array that contains a null terminated c-string with the comment to be registered. |
    |  | *structurenumber* | is an integer that specifies to which structure the comment should be added. |

**Returns:**
:   An integer that contains an error code, where 0 is no error and non-zero is an error.

|  |  |  |  |  |
| --- | --- | --- | --- | --- |
| int RNA::BreakPseudoknot | ( | const bool | *minimum\_energy* = `true`, |  |
|  |  | const int | *structurenumber* = `0` |  |
|  | ) |  |  |  |

Break any pseudoknots that might be in a structure.

This function uses the method of Smit et al. to break pseudoknots by running a dynamic programming algorithm that cannot predict pseudoknots while only allowing the pairs that already exist in the structure. When minimum\_energy = true (the default), this function predicts the lowest free energy structure that has no pseudoknots. Note that when minimum\_energy = true, this function might additionally break pairs that are not pseudoknotted if the pairs increase the folding free energy change or are forbidden (as in an isolated pair). Also note that when minum\_energy=true, this function uses the GenerateAllSuboptimalStructures methodology behind the scenes, so large internal loops in the input would lead to a loss of pairs. When minumum\_energy is set to false, this function maximizes the number of base pairs in the pseudoknot free structure. Return 0 if no error and non-zero errors can be parsed by GetErrorMessage() or GetErrorMessageString().

**Parameters:**
:   |  |  |  |
    | --- | --- | --- |
    |  | *minimum\_energy* | is a bool thgat indicates where the structure should be minimum in free energy (true) or maximize pairs (false). |
    |  | *structurenumber* | is an int that indicates a specific structure for which to break pseudoknots (indexed from 1). The default value, 0, indicates that all structures should have pseudoknots broken. |

**Returns:**
:   an int that provides an error code. 0 = no error.

|  |  |  |  |  |
| --- | --- | --- | --- | --- |
| double RNA::CalculateFreeEnergy | ( | const int | *structurenumber* = `1`, |  |
|  |  | const bool | *UseSimpleMBLoopRules* = `false` |  |
|  | ) |  |  |  |

Return the predicted Gibb's free energy change for structure # structurenumber, defaulted to 1.

Free energies are in kcal/mol. The first time this is called, if no other free energy calculation has been performed and the folding temperature has not been specifed, thermodynamic parameter files (.dat) files will be read from disk. The parameter files should be located in the directory specified by environment variable $DATAPATH, or the pwd. In case of error, the function returns a free energy change of zero. Note!: That a free energy change of zero is also a valid folding free energy change. Errors will also generate an internal error code, accessible with GetErrorCode(). GetErrorCode() will return 0 when there is no error and other codes can be parsed by GetErrorMessage() or GetErrorMessageString().

**Parameters:**
:   |  |  |  |
    | --- | --- | --- |
    |  | *structurenumber* | is an integer that refers to the index of the structure for which to calculate the folding free energy change. This defaults to 1. |
    |  | *UseSimpleMBLoopRules* | is a bool that indicates what energy rules to use. The default, false, uses the complete nearest neighbor model for multibranch loops. When true is passed, the energy model is instead a simplified model that is the one used by the dynamic programming algorithms. |

**Returns:**
:   A double which is the folding free energy change in kcal/mol.

|  |  |  |  |  |  |
| --- | --- | --- | --- | --- | --- |
| bool RNA::ContainsPseudoknot | ( | const int | *structurenumber* | ) |  |

Report if there are any pseudoknots in a structure.

This method checks for any "crossing pairs," i.e. i-j and i'-j' s.t. i < i' < j < jp. If there is at least one crossing pair set, then there is a pseudoknot and the function returns true. This function generates internal error codes that can be accessed by GetErrorCode() after the constructor is called: 0 = no error, nonzero = error. The errorcode can be resolved to a c string using GetErrorMessage.

**Parameters:**
:   |  |  |  |
    | --- | --- | --- |
    |  | *structurenumber* | is an int that indicates the structure number to check. Note that indexing of structures starts with structure #1. |

**Returns:**
:   A bool that indicates whether there is a pseudoknot.

|  |  |  |  |  |
| --- | --- | --- | --- | --- |
| int RNA::DetermineDrawingCoordinates | ( | const int | *height*, |  |
|  |  | const int | *width*, |  |
|  |  | const int | *structurenumber* = `1` |  |
|  | ) |  |  |  |

Determine the coordinates for drawing a secondary structure.

This function determines drawing coordinates for all nucleotides in structure number structurenumber. User must specify the height and width of a character (use the largest of nucleotides). The coordinates are in an abstract palette; the user must determine the minimum and maximum coordinate in both the x and y direction. The actual coordinates are fetched using GetNucleotideXCoordinate(int i) and GetNucleotideYCoordinate(int i). This function returns are error code, where 0=no error and other messages can be resolved to a c string using GetErrorMessage. The structure to be drawn must be free of pseudoknots.

**Parameters:**
:   |  |  |  |
    | --- | --- | --- |
    |  | *height* | is an integer that refers to the height of a nucleotide. |
    |  | *width* | is an integer that refers to the width of a nucleotide, where the largest nucleotide should be provided or a non-proportional font should be used. |
    |  | *structurenumber* | is an integer that refers to the structure to be drawn. |

**Returns:**
:   An int that provides an error code, 0 = no error and other errors can be resolved to a c string using GetErrorMessage.

|  |  |  |  |  |
| --- | --- | --- | --- | --- |
| int RNA::FileReader | ( | const char | *filename*[], |  |
|  |  | const int | *type* |  |
|  | ) |  |  | `[protected]` |

|  |  |  |  |  |
| --- | --- | --- | --- | --- |
| int RNA::FoldSingleStrand | ( | const float | *percent*, |  |
|  |  | const int | *maximumstructures*, |  |
|  |  | const int | *window*, |  |
|  |  | const char | *savefile*[] = `""`, |  |
|  |  | const int | *maxinternalloopsize* = `30`, |  |
|  |  | bool | *mfeonly* = `false` |  |
|  | ) |  |  |  |

Predict the lowest free energy secondary structure and generate suboptimal structures using a heuristic.

This function predicts the lowest free energy structure and suboptimal structures. If the temperature has not been specified using SetTemperature and no free energies have been calculated, the thermodynamic parameters have not been read and therefore they will be read by this function call. The parameter files should be located in the directory specified by the environment variable $DATAPATH of the pwd. In case of error, the function returns a non-zero that can be parsed by GetErrorMessage() or GetErrorMessageString().

**Parameters:**
:   |  |  |  |
    | --- | --- | --- |
    |  | *percent* | is the maximum % difference in free energy in suboptimal structures from the lowest free energy structure. |
    |  | *maximumstructures* | is the maximum number of suboptimal structures to generate. |
    |  | *window* | is a parameter that specifies how different the suboptimal structures should be from each other (0=no restriction and larger integers require structures to be more different). |
    |  | *savefile* | is c string containing a file path and name for a savefile (.sav)that can be used to generate energy dot plots and to refold the secondary structure using different suboptimal structure parameters. The default is "", which results in no save file written. |
    |  | *maxinternalloopsize* | is the maximum number of unpaired nucleotides in bulge and internal loops. This is used to accelerate the prediction speed. The default is 30. |
    |  | *mfeonly* | is a bool that indicates whether only the minimum free energy structure will be generated. This saves half the calculation time, but no save file can be generated. Default is false. |

**Returns:**
:   An int that indicates an error code (0 = no error, 5 = error reading thermodynamic parameter files, 14 = traceback error).

|  |  |  |  |  |  |
| --- | --- | --- | --- | --- | --- |
| int RNA::ForceDoubleStranded | ( | const int | *i* | ) |  |

Force a nucleotide to be double stranded (base paired).

This function indicates a nucleotide that is double stranded (paired). In subsequent structure prediction, this nucleotide will be double stranded. The function returns 0 with no error and a non-zero otherwise that can be parsed by GetErrorMessage() or GetErrorMessageString().

**Parameters:**
:   |  |  |  |
    | --- | --- | --- |
    |  | *i* | is the index of the paired nucleotide. |

**Returns:**
:   An integer that indicates an error code (0 = no error, 4 = nucleotide out of range, 8 = too many restraints specified, 9 = same nucleotide in conflicting restraint).

|  |  |  |  |  |  |
| --- | --- | --- | --- | --- | --- |
| int RNA::ForceFMNCleavage | ( | const int | *i* | ) |  |

Indicate a nucleotide that is accessible to FMN cleavage (a U in GU pair).

In subsequent structure prediction, this nucleotide will be in a GU pair. The function returns 0 with no error and a non-zero otherwise that can be parsed by GetErrorMessage() or GetErrorMessageString().

**Parameters:**
:   |  |  |  |
    | --- | --- | --- |
    |  | *i* | is the index of the FMN-cleaved nucleotide. |

**Returns:**
:   An integer that indicates an error code (0 = no error, 4 = nucleotide out of range, 8 = too many restraints specified, 9 = same nucleotide in conflicting restraint, 11 = nucleotide not U).

|  |  |  |  |  |  |
| --- | --- | --- | --- | --- | --- |
| int RNA::ForceMaximumPairingDistance | ( | const int | *distance* | ) |  |

Force a maximum distance between apired nucleotides.

In a subsequent structure prediction, there will be no pairs allowed between nucleotides more distant than distance, i.e. |j-i| < distance for i to pair to j. The function returns and error code; 0==no error, 12== too long or too short distance.

**Parameters:**
:   |  |  |  |
    | --- | --- | --- |
    |  | *distance* | is the maximum pairing distance. |

**Returns:**
:   An integer that indicates an error code (0 = no error, 12 = too short).

|  |  |  |  |  |  |
| --- | --- | --- | --- | --- | --- |
| int RNA::ForceModification | ( | const int | *i* | ) |  |

Force modification for a nucleotide.

This function indicates a nucleotide that is accessible to chemical modification. In subsequent structure prediction, this nucleotide will be single stranded, at the end of a helix, or in or adjacent to a GU pair. The function returns 0 with no error and a non-zero otherwise that can be parsed by GetErrorMessage() or GetErrorMessageString().

**Parameters:**
:   |  |  |  |
    | --- | --- | --- |
    |  | *i* | is the index of the nucleotide accessible to chemical modification. |

**Returns:**
:   An integer that indicates an error code (0 = no error, 4 = nucleotide out of range, 8 = too many restraints specified).

|  |  |  |  |  |
| --- | --- | --- | --- | --- |
| int RNA::ForcePair | ( | const int | *i*, |  |
|  |  | const int | *j* |  |
|  | ) |  |  |  |

Force a pair between two nucleotides.

This function forces a pair between two nucleotides in subsequent structure predictions. When multiple pairs are specified, the pairs must not force a pseudoknot. The function returns 0 with no error and a non-zero otherwise that can be parsed by GetErrorMessage() or GetErrorMessageString().

**Parameters:**
:   |  |  |  |
    | --- | --- | --- |
    |  | *i* | is the index of one nucleotide in the pair. |
    |  | *j* | is the index of the second nucleotide in the pair. |

**Returns:**
:   An integer that indicates an error code (0 = no error, 4 = nucleotide out of range, 6 = pseudoknot formation, 7 = non-canonical pair, 8 = too many restraints specified, 9 = same nucleotide in conflicting restraint).

|  |  |  |  |  |
| --- | --- | --- | --- | --- |
| int RNA::ForceProhibitPair | ( | const int | *i*, |  |
|  |  | const int | *j* |  |
|  | ) |  |  |  |

Prohibit a pair between two nucleotides.

This function prevents a pair between two nucleotides in subsequent structure predictions. The function returns 0 with no error and a non-zero otherwise that can be parsed by GetErrorMessage() or GetErrorMessageString().

**Parameters:**
:   |  |  |  |
    | --- | --- | --- |
    |  | *i* | is the index of one nucleotide in the pair. |
    |  | *j* | is the index of the second nucleotide in the pair. |

**Returns:**
:   An integer that indicates an error code (0 = no error, 4 = nucleotide out of range, 8 = too many restraints specified, 9 = nucleotide in conflicting restraint).

|  |  |  |  |  |  |
| --- | --- | --- | --- | --- | --- |
| int RNA::ForceSingleStranded | ( | const int | *i* | ) |  |

Force a nucleotide to be single stranded.

This function indicates a nucleotide that is single stranded. In subsequent structure prediction, this nucleotide will be single stranded. The function returns 0 with no error and a non-zero otherwise that can be parsed by GetErrorMessage() or GetErrorMessageString().

**Parameters:**
:   |  |  |  |
    | --- | --- | --- |
    |  | *i* | is the index of the nucleotide that is single stranded. |

**Returns:**
:   An integer that indicates an error code (0 = no error, 4 = nucleotide out of range, 8 = too many restraints specified, 9 = same nucleotide in conflicting restraint).

|  |  |  |  |  |
| --- | --- | --- | --- | --- |
| int RNA::GenerateAllSuboptimalStructures | ( | const float | *percent*, |  |
|  |  | const double | *deltaG* |  |
|  | ) |  |  |  |

Predict the lowest free energy secondary structure and generate all suboptimal structures.

This function predicts the lowest free energy structure and suboptimal structures. If the temperature has not been specified using SetTemperature and no free energies have been calculated, the thermodynamic parameters have not been read and therefore they will be read by this function call. The parameter files should be located in the directory specified by the environment variable $DATAPATH of the pwd. In case of error, the function returns a non-zero that can be parsed by GetErrorMessage() or GetErrorMessageString(). Two controls are available for limiting the number of structures, the maximum % difference in energy (percent) and the maximum absolute change in energy (deltaG). The smaller of the two will be used as the limit.

**Parameters:**
:   |  |  |  |
    | --- | --- | --- |
    |  | *percent* | is the maximum % difference in free energy in suboptimal structures from the lowest free energy structure. |
    |  | *deltaG* | is the maximum difference in free energy change above the lowest free energy structure (in kcal/mol). |

**Returns:**
:   An int that indicates an error code (0 = no error, non-zero = error).

|  |  |  |  |  |
| --- | --- | --- | --- | --- |
| bool RNA::GetBackboneType | ( |  | ) |  |

Get the backbone type.

This function returns whether the backbone is RNA or DNA. Note that backbone type is set when calling the constructor.

**Returns:**
:   A bool that indicates the backbone (true = RNA, false = DNA).

|  |  |  |  |  |  |
| --- | --- | --- | --- | --- | --- |
| std::string RNA::GetCommentString | ( | const int | *structurenumber* = `1` | ) |  |

Provide the comment from the ct file as a string.

This function provides the comment from the CT file for a structure as a string. This function generates internal error codes that can be accessed by GetErrorCode() after the function is called: 0 = no error, nonzero = error. The errorcode can be resolved to a c string using GetErrorMessage.

**Parameters:**
:   |  |  |  |
    | --- | --- | --- |
    |  | *structurenumber* | is the structure for which the comment is to be provided. |

**Returns:**
:   A string that provides the comment.

|  |  |  |  |  |
| --- | --- | --- | --- | --- |
| double RNA::GetEnsembleEnergy | ( |  | ) |  |

Get the ensemble folding free energy change.

Returns the ensemble folding free energy change as determined by the partition function. This is a handy way of getting the size of the partition function Q, which itself is too large to fit in a double for all but the shortest sequences. The ensemble folding free energy change = -RT ln (Q). Function requires that the partition function data be present either because PartitionFunction() has been called or the constructor that reads a partition function save was used. This function generates internal error codes that can be accessed by GetErrorCode() after the constructor is called: 0 = no error, nonzero = error. The errorcode can be resolved to a c string using GetErrorMessage.

**Returns:**
:   A double that is the ensemble folding free energy change in kcal/mol.

|  |  |  |  |  |
| --- | --- | --- | --- | --- |
| int RNA::GetErrorCode | ( |  | ) |  |

Return an error code, where a return of zero is no error.

This function returns and error flag that is generated during construction by RNA(const char &filename, const int type, const bool IsRNA=true) or from CalculateFreeEnergy(). An error of zero is always no error. Other codes are errors and a c-string can be fetched for the error with GetErrorMessage().

**Returns:**
:   An integer that provides the error code.

Reimplemented in HybridRNA.

|  |  |  |  |  |  |
| --- | --- | --- | --- | --- | --- |
| char \* RNA::GetErrorMessage | ( | const int | *error* | ) |  |

Return error messages based on code from GetErrorCode and other error codes.

0 = no error 1 = input file not found 2 = error opening file 3 = structure number out of range 4 = nucleotide number out of range 5 = error reading thermodynamic parameters 6 = pseudoknot formation 7 = non-canonical pair 8 = too many restraints specified 9 = same nucleotide in conflicting restraint 10 = no structures to write 11 = nucleotide not a U (caused by ForceFMNCleavage() 12 = distance too short 13 = error reading constraint file 14 = traceback error 15 = no partition function data present 16 = incorrect save file version used 17 = cannot be performed without having read a save file (.sav) 18 = threshold is too low to be valid 19 = drawing coordinates have not been determined 20 = no sequence has been read 21 = over 1 probability error on stochastic traceback 22 = programming error, unrecognized input to constructor 23 = no structures present 24 = too few iterations 25 = index (for drawing) is not a multiple of 10

**Parameters:**
:   |  |  |  |
    | --- | --- | --- |
    |  | *error* | is the integer error code provided by GetErrorCode() or from other functions that return integer error codes. |

**Returns:**
:   A pointer to a c string that provides an error message.

Reimplemented in HybridRNA, and Oligowalk\_object.

|  |  |  |  |  |  |
| --- | --- | --- | --- | --- | --- |
| std::string RNA::GetErrorMessageString | ( | const int | *error* | ) |  |

Return error messages based on code from GetErrorCode and other error codes.

Although RNA generally uses c strings, this member function returns a string that is suitable for interfacing with JAVA, etc. See the error list in the GetErrorMessage() entry.

**Parameters:**
:   |  |  |  |
    | --- | --- | --- |
    |  | *error* | is the integer error code provided by GetErrorCode() or from other functions that return integer error codes. |

**Returns:**
:   A string that provides an error message.

|  |  |  |  |  |  |
| --- | --- | --- | --- | --- | --- |
| int RNA::GetForcedDoubleStranded | ( | const int | *constraintnumber* | ) |  |

Return a nucleotide that is forced double stranded.

This function returns a nucleotide that is constrainted to be paired. Constraints are numbered from zero to GetNumberofForcedDoubleStranded()-1.

**Parameters:**
:   |  |  |  |
    | --- | --- | --- |
    |  | *constraintnumber* | is the index to the constraint number. |

**Returns:**
:   An integer that is the nucleotide index. If the constraintnumber is for a constraint that does not exist, zero is returned.

|  |  |  |  |  |  |
| --- | --- | --- | --- | --- | --- |
| int RNA::GetForcedFMNCleavage | ( | const int | *constraintnumber* | ) |  |

Return a nucleotide that is accessible to FMN cleavage.

This function returns a nucleotide that is constrainted to be accessible to FMN cleavage (a U in a GU pair). Constraints are numbered from zero to GetNumberofForcedFMNCleavages()-1.

**Parameters:**
:   |  |  |  |
    | --- | --- | --- |
    |  | *constraintnumber* | is the index to the constraint number. |

**Returns:**
:   An integer that is the nucleotide index. If the constraintnumber is for a constraint that does not exist, zero is returned.

|  |  |  |  |  |  |
| --- | --- | --- | --- | --- | --- |
| int RNA::GetForcedModification | ( | const int | *constraintnumber* | ) |  |

Return a nucleotide that is accessible to modification.

This function returns a nucleotide that is constrainted to be accessible to chemical modification. Constraints are numbered from zero to GetNumberofModifications()-1.

**Parameters:**
:   |  |  |  |
    | --- | --- | --- |
    |  | *constraintnumber* | is the index to the constraint number. |

**Returns:**
:   An integer that is the nucleotide index. If the constraintnumber is for a constraint that does not exist, zero is returned.

|  |  |  |  |  |
| --- | --- | --- | --- | --- |
| int RNA::GetForcedPair | ( | const int | *constraintnumber*, |  |
|  |  | const bool | *fiveprime* |  |
|  | ) |  |  |  |

Return a nucleotide in a forced pair.

This function returns either the five prime or three prime nucleotide in a forced pair constraint, depending on the value of fiveprime. Constraints are numbered from zero to GetNumberofForcedPairs()-1.

**Parameters:**
:   |  |  |  |
    | --- | --- | --- |
    |  | *constraintnumber* | is the index to the constraint number. |
    |  | *fiveprime* | determines if the nucleotide is the five prime or the three prime nucleotide in the constraint. true = five prime nucleotide. |

**Returns:**
:   An integer that is the nucleotide index. If the constraintnumber is for a constraint that does not exist, zero is returned.

|  |  |  |  |  |
| --- | --- | --- | --- | --- |
| int RNA::GetForcedProhibitedPair | ( | const int | *constraintnumber*, |  |
|  |  | const bool | *fiveprime* |  |
|  | ) |  |  |  |

Return a nucleotide in a prohibited pair.

This function returns either the five prime or three prime nucleotide in a prohibited pair constraint, depending on the value of fiveprime. Constraints are numbered from zero to GetNumberofForcedProhibited()-1.

**Parameters:**
:   |  |  |  |
    | --- | --- | --- |
    |  | *constraintnumber* | is the index to the constraint number. |
    |  | *fiveprime* | determines if the nucleotide is the five prime or the three prime nucleotide in the constraint. true = five prime nucleotide. |

**Returns:**
:   An integer that is the nucleotide index. If the constraintnumber is for a constraint that does not exist, zero is returned.

|  |  |  |  |  |  |
| --- | --- | --- | --- | --- | --- |
| int RNA::GetForcedSingleStranded | ( | const int | *constraintnumber* | ) |  |

Return a nucleotide that is forced single stranded.

This function returns a nucleotide that is constrainted to be single stranded. Constraints are numbered from zero to GetNumberofForcedSingleStranded()-1.

**Parameters:**
:   |  |  |  |
    | --- | --- | --- |
    |  | *constraintnumber* | is the index to the constraint number. |

**Returns:**
:   An integer that is the nucleotide index. If the constraintnumber is for a constraint that does not exist, zero is returned.

|  |  |  |  |  |  |
| --- | --- | --- | --- | --- | --- |
| double RNA::GetFreeEnergy | ( | const int | *structurenumber* | ) |  |

Get the folding free energy change for a predicted structure.

Returns the folding free energy change of structure i as determined by a previous folding calculation. Function requires that the structure be predicted by a structure prediction method. This function generates internal error codes that can be accessed by GetErrorCode() after the constructor is called: 0 = no error, nonzero = error. The errorcode can be resolved to a c string using GetErrorMessage.

**Parameters:**
:   |  |  |  |
    | --- | --- | --- |
    |  | *structurenumber* | is an integer indicating the predicted structure number. |

**Returns:**
:   A double that is the folding free energy change in kcal/mol.

|  |  |  |  |  |  |
| --- | --- | --- | --- | --- | --- |
| int RNA::GetLabelXCoordinate | ( | const int | *i* | ) |  |

Get the X coordinate for placing the nucleotide index label specified by i.

This function gets the X coordinate for placing the nucleotide index label specified by i. The user needs to have determined the coordinates for a complete structure using DetermineDrawingCoordinates prior to making this call. This function generates internal error codes that can be accessed by GetErrorCode(): 0 = no error, nonzero = error. The errorcode can be resolved to a c string using GetErrorMessage. Zero is returned in case of error, but note that zero is also a valid coordinate. One additiona caveat: Labels that are placed at 0,0 are lables that would have overlapped nucleotides. These labels should not be drawn.

**Parameters:**
:   |  |  |  |
    | --- | --- | --- |
    |  | *i* | is an integer refering to the label to be drawn. This needs to be a multiple of 10. |

**Returns:**
:   An int that gives the X coordinate.

|  |  |  |  |  |  |
| --- | --- | --- | --- | --- | --- |
| int RNA::GetLabelYCoordinate | ( | const int | *i* | ) |  |

Get the Y coordinate for placing the nucleotide index label specified by i.

This function gets the Y coordinate for placing the nucleotide index label specified by i. The user needs to have determined the coordinates for a complete structure using DetermineDrawingCoordinates prior to making this call. This function generates internal error codes that can be accessed by GetErrorCode(): 0 = no error, nonzero = error. The errorcode can be resolved to a c string using GetErrorMessage. Zero is returned in case of error, but note that zero is also a valid coordinate. One additiona caveat: Labels that are placed at 0,0 are lables that would have overlapped nucleotides. These labels should not be drawn.

**Parameters:**
:   |  |  |  |
    | --- | --- | --- |
    |  | *i* | is an integer refering to the label to be drawn. This needs to be a multiple of 10. |

**Returns:**
:   An int that gives the Y coordinate.

|  |  |  |  |  |
| --- | --- | --- | --- | --- |
| int RNA::GetMaximumPairingDistance | ( |  | ) |  |

Return the maximum pairing distance.

return An integer that indicates the maximum distance allowed between paired nucleotides, where -1 indicates that the maximum distance is not set.

|  |  |  |  |  |  |
| --- | --- | --- | --- | --- | --- |
| char RNA::GetNucleotide | ( | const int | *i* | ) |  |

param An integer specifying the nucleotide index (starting at 1 and ending at GetSequenceLength()). This function generates internal error codes that can be accessed by GetErrorCode(): 0 = no error, nonzero = error. The errorcode can be resolved to a c string using GetErrorMessage. Note that nucleotides are numbered starting at an index of 1. return The char representing the nucleotide at index i or '-' if an error occured.

|  |  |  |  |  |  |
| --- | --- | --- | --- | --- | --- |
| int RNA::GetNucleotideXCoordinate | ( | const int | *i* | ) |  |

Get the X coordinate for nucleotide i for drawing a structure.

This function gets the X coordinate for placing the nucleotide specified by i. The user needs to have determined the coordinates for a complete structure using DetermineDrawingCoordinates prior to making this call. This function generates internal error codes that can be accessed by GetErrorCode(): 0 = no error, nonzero = error. The errorcode can be resolved to a c string using GetErrorMessage. Zero is returned in case of error, but note that zero is also a valid coordinate.

**Parameters:**
:   |  |  |  |
    | --- | --- | --- |
    |  | *i* | is an integer refering to the nucleotide to be drawn. |

**Returns:**
:   An int that gives the X coordinate.

|  |  |  |  |  |  |
| --- | --- | --- | --- | --- | --- |
| int RNA::GetNucleotideYCoordinate | ( | const int | *i* | ) |  |

Get the Y coordinate for nucleotide i for drawing a structure.

This function gets the Y coordinate for placing the nucleotide specified by i. The user needs to have determined the coordinates for a complete structure using DetermineDrawingCoordinates prior to making this call. This function generates internal error codes that can be accessed by GetErrorCode(): 0 = no error, nonzero = error. The errorcode can be resolved to a c string using GetErrorMessage. Zero is returned in case of error, but note that zero is also a valid coordinate.

**Parameters:**
:   |  |  |  |
    | --- | --- | --- |
    |  | *i* | is an integer refering to the nucleotide to be drawn. |

**Returns:**
:   An int that gives the Y coordinate.

|  |  |  |  |  |
| --- | --- | --- | --- | --- |
| int RNA::GetNumberOfForcedDoubleStranded | ( |  | ) |  |

Return the number of nucletides forced to be paired.

**Returns:**
:   An integer that indicates the number of nucleotides that are forced pair.

|  |  |  |  |  |
| --- | --- | --- | --- | --- |
| int RNA::GetNumberOfForcedFMNCleavages | ( |  | ) |  |

Return the number of nucleotides accessible to FMN cleavage.

**Returns:**
:   An integer that indicates the number of FMN cleavage nucleotides (Us in GU pairs).

|  |  |  |  |  |
| --- | --- | --- | --- | --- |
| int RNA::GetNumberOfForcedModifications | ( |  | ) |  |

Return the number of nucleotides accessible to chemical modification.

**Returns:**
:   An integer that indicates the number of modified nucleotides.

|  |  |  |  |  |
| --- | --- | --- | --- | --- |
| int RNA::GetNumberOfForcedPairs | ( |  | ) |  |

Return the number of forced base pairs.

**Returns:**
:   An integer that indicates the number of forced pairs.

|  |  |  |  |  |
| --- | --- | --- | --- | --- |
| int RNA::GetNumberOfForcedProhibitedPairs | ( |  | ) |  |

Return the number of prohibited base pairs.

**Returns:**
:   An integer that indicates the number of pairs that are prohibited.

|  |  |  |  |  |
| --- | --- | --- | --- | --- |
| int RNA::GetNumberOfForcedSingleStranded | ( |  | ) |  |

Return the number of nucleotides that are not allowed to pair.

**Returns:**
:   An integer that indicates the number of nucleotides not allowed to pair.

|  |  |  |  |  |
| --- | --- | --- | --- | --- |
| int RNA::GetPair | ( | const int | *i*, |  |
|  |  | const int | *structurenumber* = `1` |  |
|  | ) |  |  |  |

Get the nucleotide to which the specified nucleotide is paired.

Returns the pairing partner of the ith nucleotide in structure number structurenumber. Zero means the nucleotide is unpaired. This function generates internal error codes that can be accessed by GetErrorCode() after the constructor is called: 0 = no error, nonzero = error. The errorcode can be resolved to a c string using GetErrorMessage.

**Parameters:**
:   |  |  |  |
    | --- | --- | --- |
    |  | *i* | is an int that indicates the nucleotide to which the pairing partner is being queried. |
    |  | *structurenumber* | is an int that indicates the structure number, where the default is 1. |

**Returns:**
:   An int that indicates the other nucleotide in pair, where 0 is no paired.

|  |  |  |  |  |
| --- | --- | --- | --- | --- |
| double RNA::GetPairEnergy | ( | const int | *i*, |  |
|  |  | const int | *j* |  |
|  | ) |  |  |  |

Get the lowest folding free energy possible for a structure containing pair i-j.

Returns a folding free energy change in kcal/mol for use in energy dot plots. This function requires that the RNA constructor be called with a save file (.sav) name. (That is, for historical resaons, this cannot be called after FoldSingleStrand without writing the data to disk.) This function generates internal error codes that can be accessed by GetErrorCode() after the constructor is called: 0 = no error, nonzero = error. The errorcode can be resolved to a c string using GetErrorMessage. param i and j are ints that provide indexes the 5' and 3' nucleotides, respectively, in a pair. return A double that is the folding free energy change in kcal/mol.

|  |  |  |  |  |
| --- | --- | --- | --- | --- |
| double RNA::GetPairProbability | ( | const int | *i*, |  |
|  |  | const int | *j* |  |
|  | ) |  |  |  |

Get a base pair probability.

Returns the base pair probability for the pair between i and j. Function requires that the partition function data be present either because PartitionFunction() has been called or the constructor that reads a partition function save was used. This function generates internal error codes that can be accessed by GetErrorCode(): 0 = no error, nonzero = error. The errorcode can be resolved to a c string using GetErrorMessage.

**Parameters:**
:   |  |  |  |
    | --- | --- | --- |
    |  | *i* | provides the 5' nucleotide in a pair. |
    |  | *j* | provides the 3' nucleotides in a pair. |

**Returns:**
:   A double that is the base pair probability. If i and j cannot pair, 0.0 is returned. If an error occurs, 0.0 is returned.

|  |  |  |  |  |
| --- | --- | --- | --- | --- |
| TProgressDialog \* RNA::GetProgress | ( |  | ) |  |

Return the current pointer to TProgressDialog. This is used during inheritance to provide access to the underlying TProgressDialog.

|  |  |  |  |  |
| --- | --- | --- | --- | --- |
| int RNA::GetSequenceLength | ( |  | ) |  |

Get the total length of the sequence.

**Returns:**
:   An integer that specifies the total length of the sequence.

|  |  |  |  |  |
| --- | --- | --- | --- | --- |
| structure \* RNA::GetStructure | ( |  | ) |  |

Access the underlying structure class. This is provided for use with two sequence methods. Generally, there is no need for end users to use this function because the RNA class provides an convenient wrapper for accessing the information in an RNA class.

**Returns:**
:   A pointer to structure.

|  |  |  |  |  |
| --- | --- | --- | --- | --- |
| int RNA::GetStructureNumber | ( |  | ) |  |

Get the total number of specified or predicted structures.

**Returns:**
:   An integer specify the total number of structures.

|  |  |  |  |  |
| --- | --- | --- | --- | --- |
| int RNA::MaximizeExpectedAccuracy | ( | const double | *maxPercent*, |  |
|  |  | const int | *maxStructures*, |  |
|  |  | const int | *window*, |  |
|  |  | const double | *gamma* = `1.0` |  |
|  | ) |  |  |  |

Predict the structure with maximum expected accuracy and suboptimal structures.

This function predicts structures composed of probable base pairs and single-srtranded nucleotide, weighted by gamma. The score for a structure is = gamma \* 2 \* (sum of pairing probabilities for pairs) + (sum of unpairing probabilities for single stranded nucleotides). This function requires partition function data from either a previous partition function calculations or from having read a partition function save file during construction of the class. In case of error, the function returns a non-zero that can be parsed by GetErrorMessage() or GetErrorMessageString().

**Parameters:**
:   |  |  |  |
    | --- | --- | --- |
    |  | *maxPercent* | is the maximum percent difference is score in generating suboptimal structures. |
    |  | *maxStructures* | is the maximum number of suboptimal structures to generate. |
    |  | *window* | is the window parameter, where a higher value generates suboptimal structures that are more different from each other. |
    |  | *gamma* | is the weight given to base pairs |

**Returns:**
:   An int that indicates an error code (0 = no error, non-zero = error).

|  |  |  |  |  |
| --- | --- | --- | --- | --- |
| int RNA::PartitionFunction | ( | const char | *savefile*[] = `""`, |  |
|  |  | double | *temperature* = `-10.0` |  |
|  | ) |  |  |  |

Predict the partition function for a sequence.

This function must be called to predict base pair probabilities, perform stochastic traceback, or for maximizing expected accuracy. If the temperature has not been specified using SetTemperature and no free energies have been calculated, the thermodynamic parameters have not been read and therefore they will be read by this function call. The parameter files should be located in the directory specified by the environment variable $DATAPATH of the pwd. In case of error, the function returns a non-zero that can be parsed by GetErrorMessage() or GetErrorMessageString(). Note that the parameter temperature is used when calculating equilibrium constants, but does not change the temperature at which the free energies are determined. SetTemperature, from the underlying base class Thermodynamics, should be used to change the temperature for most calculations. This parameter should generally not be used. The default is -10.0 and values below zero cause this parameter to be ignored (the correct default behavior). Note also that if SetTemperature is not used, the temperature defaults to 310.15 K (37 deg. C), which is the desired behavior for most purposes.

**Parameters:**
:   |  |  |  |
    | --- | --- | --- |
    |  | *savefile* | is a c string that contains the path and filename for creating a save file. This defaults to "", which indicates no file is to be written. |
    |  | *temperature* | is a double that indicates a pseudo-temperature for calculating equilibrium constants from free energies at fixed temperature previously specified. |

**Returns:**
:   An int that indicates an error code (0 = no error, 5 = error reading thermodynamic parameter files).

|  |  |  |  |  |  |
| --- | --- | --- | --- | --- | --- |
| int RNA::PredictProbablePairs | ( | const float | *probability* = `0` | ) |  |

Predict structures containing highly probable pairs.

This function predicts structures composed of probable base pairs. This function requires partition function data from either a previous partition function calculations or from having read a partition function save file during construction of the class. In case of error, the function returns a non-zero that can be parsed by GetErrorMessage() or GetErrorMessageString().

**Parameters:**
:   |  |  |  |
    | --- | --- | --- |
    |  | *probability* | is the pairing probability threshold, where pairs will be predicted if they have a higher probability. Note that a value of less than 0.5 (50%), will cause an error. The default value of zero will trigger the creation of 8 structures, with thresholds of >=0.99, >=0.97, >=0.95, >=0.90, >=0.80, >=0.70, >=0.60, >0.50. |

**Returns:**
:   An int that indicates an error code (0 = no error, non-zero = error).

|  |  |  |  |  |
| --- | --- | --- | --- | --- |
| int RNA::ProbKnot | ( | int | *iterations* = `1`, |  |
|  |  | int | *MinHelixLength* = `1` |  |
|  | ) |  |  |  |

Predict maximum expected accuracy structures that contain pseudoknots.

This function uses base pair probabilities to predict structures that contains pseudoknots. This function requires partition function data from either a previous partition function calculations or from having read a partition function save file during construction of the class. In case of error, the function returns a non-zero that can be parsed by GetErrorMessage() or GetErrorMessageString().

**Parameters:**
:   |  |  |  |
    | --- | --- | --- |
    |  | *iterations* | is the number of iterations of pair selection that are performed. The default and recommended value is 1. |
    |  | *MinHelixLength* | is the shortest helix that is allowed. If this is set >1, a post-processing step is performed to remove short helices. Default = 1, i.e. no post-processing. |

**Returns:**
:   An int that indicates an error code (0 = no error, non-zero = error).

|  |  |  |  |  |  |
| --- | --- | --- | --- | --- | --- |
| int RNA::ReadConstraints | ( | const char | *filename*[] | ) |  |

Read a set of folding constraints to disk in a plain text file.

The file format for constraints is that generated by the WriteConstraints() function. The function returns 0 with no error and a non-zero otherwise that can be parsed by GetErrorMessage() or GetErrorMessageString(). Note that calling ReadConstraints() will erase previously defined constraints (except for SHAPE pseudoenergy restraints).

**Parameters:**
:   |  |  |  |
    | --- | --- | --- |
    |  | *filename* | is a c string that is the file name to be read. |

**Returns:**
:   An integer that indicates an error code (0 = no error, 1 = file not found, 13 = error reading constraint file).

|  |  |  |  |  |  |
| --- | --- | --- | --- | --- | --- |
| int RNA::ReadDSO | ( | const char | *filename*[] | ) |  |

Read double strand offset data from disk.

The double strand offset is data that is used to constrain structure prediction on subsequent structure predictions. This is a free energy in kcal/mol that is added to a specific nucleotide that is double stranded. The function returns 0 with no error and a non-zero otherwise that can be parsed by GetErrorMessage() or GetErrorMessageString().

**Parameters:**
:   |  |  |  |
    | --- | --- | --- |
    |  | *filename* | is a c string that indicates a file that contains data, in a raw format with nucleotide index and offset (one set per line). |

**Returns:**
:   An integer that indicates an error code (0 = no error, 1 = input file not found).

|  |  |  |  |  |
| --- | --- | --- | --- | --- |
| int RNA::ReadExperimentalPairBonus | ( | const char | *filename*[], |  |
|  |  | double const | *experimentalOffset*, |  |
|  |  | double const | *experimentalScaling* |  |
|  | ) |  |  |  |

Read experimental pair bonuses from disk.

This is a quantity that results in a bonus added to a specific pair, once per stack, so that pairs in the middle of a helix get the bonus twice and those at the end of a helix get the bonus once. The bonus is in the form of experimentalScaling\*value + experimentalOffset. The data is formatted using a simple square matrix of values and no headers. The format requires that there be N^2 entries for a sequence of N nucleotides.

**Parameters:**
:   |  |  |  |
    | --- | --- | --- |
    |  | *filename* | is a c string that indicates a file that contains data. |
    |  | *experimentalOffset* | is a double that is added to each value. |
    |  | *experimentalScaling* | is a double by which each value is multiplied. |

**Returns:**
:   An integer that indicates an error code (0 = no error, 1 = input file not found).

|  |  |  |  |  |
| --- | --- | --- | --- | --- |
| int RNA::ReadSHAPE | ( | const char | *filename*[], |  |
|  |  | const double | *parameter1*, |  |
|  |  | const double | *parameter2*, |  |
|  |  | const double | *ssm*, |  |
|  |  | const double | *ssb*, |  |
|  |  | std::string | *modifier* = `"SHAPE"` |  |
|  | ) |  |  |  |

Read SHAPE data from disk including single-stranded SHAPE pseudo free energys.

The SHAPE data is used to constrain structure prediction on subsequent structure predictions. This version of the overloaded function includes a single-stranded pseudo free energy change. The function returns 0 with no error and a non-zero otherwise that can be parsed by GetErrorMessage() or GetErrorMessageString(). Pseudo folding free energy change parameters should be in units of kcal/mol.

**Parameters:**
:   |  |  |  |
    | --- | --- | --- |
    |  | *filename* | is a c string that indicates a file that contains SHAPE data. |
    |  | *parameter1* | is the double-stranded slope. |
    |  | *parameter2* | is the double-stranded intercept. |
    |  | *modifier* | is the type of chemical modification probe that was used (currently accepted values are SHAPE, DMS, and CMCT). Defaults to SHAPE. |
    |  | *ssm* | is the single-stranded slope. |
    |  | *ssb* | is the single-stranded intercept. |

**Returns:**
:   An integer that indicates an error code (0 = no error, 1 = input file not found).

|  |  |  |  |  |
| --- | --- | --- | --- | --- |
| int RNA::ReadSHAPE | ( | const char | *filename*[], |  |
|  |  | const double | *parameter1*, |  |
|  |  | const double | *parameter2*, |  |
|  |  | std::string | *modifier* = `"SHAPE"`, |  |
|  |  | const bool | *IsPseudoEnergy* = `true` |  |
|  | ) |  |  |  |

Read SHAPE data from disk.

The SHAPE data is used to constrain structure prediction on subsequent structure predictions. The function returns 0 with no error and a non-zero otherwise that can be parsed by GetErrorMessage() or GetErrorMessageString(). Pseudo folding free energy change parameters should be in units of kcal/mol.

**Parameters:**
:   |  |  |  |
    | --- | --- | --- |
    |  | *filename* | is a c string that indicates a file that contains SHAPE data. |
    |  | *IsPseudoEnergy* | indicates whether this is the pseudo folding free energy constraint (the preferred method). This defaults to true. |
    |  | *parameter1* | is the slope when IsPseudoEnergy=true and is a threshold above which nucleotides are forced single stranded otherwise. |
    |  | *parameter2* | is the intercept when IsPseudoEnergy=true and is a threshold above which a nucleotide is considered chemically modified otherwise. |
    |  | *modifier* | is the type of chemical modification probe that was used (currently accepted values are SHAPE, DMS, and CMCT). Defaults to SHAPE. |

**Returns:**
:   An integer that indicates an error code (0 = no error, 1 = input file not found).

|  |  |  |  |  |  |
| --- | --- | --- | --- | --- | --- |
| int RNA::ReadSSO | ( | const char | *filename*[] | ) |  |

Read single strand offset data from disk.

The single strand offset is data that is used to constrain structure prediction on subsequent structure predictions. This is a free energy in kcal/mol that is added to a specific nucleotide that is single stranded. The function returns 0 with no error and a non-zero otherwise that can be parsed by GetErrorMessage() or GetErrorMessageString().

**Parameters:**
:   |  |  |  |
    | --- | --- | --- |
    |  | *filename* | is a c string that indicates a file that contains data, in a raw format with nucleotide index and offset (one set per line). |

**Returns:**
:   An integer that indicates an error code (0 = no error, 1 = input file not found).

|  |  |  |  |  |
| --- | --- | --- | --- | --- |
| int RNA::ReFoldSingleStrand | ( | const float | *percent*, |  |
|  |  | const int | *maximumstructures*, |  |
|  |  | const int | *window* |  |
|  | ) |  |  |  |

Re-predict the lowest free energy secondary structure and generate suboptimal structures using a heuristic.

This function predicts the lowest free energy structure and suboptimal structure after a save file (.sav) was specified to the constructor. The step of predicting structures from the save file is rapid, so this is laregely a method to quickly generate a different set of suboptimal structures. Refolding can only be performed if the RNA constructor was called with a save file name. (That is, you cannot call this after calling fold single strand, without loading the data from disk with a new instance of RNA. This is for historical reasons.) In case of error, the function returns a non-zero that can be parsed by GetErrorMessage() or GetErrorMessageString().

**Parameters:**
:   |  |  |  |
    | --- | --- | --- |
    |  | *percent* | is the maximum % difference in free energy in suboptimal structures from the lowest free energy structure. |
    |  | *maximumstructures* | is the maximum number of suboptimal structures to generate. |
    |  | *window* | is a parameter that specifies how different the suboptimal structures should be from each other (0=no restriction and larger integers require structures to be more different). |

**Returns:**
:   An int that indicates an error code (0 = no error, 5 = error reading thermodynamic parameter files, 14 = traceback error).

|  |  |  |  |  |
| --- | --- | --- | --- | --- |
| int RNA::RemoveBasePair | ( | const int | *i*, |  |
|  |  | const int | *structurenumber* = `1` |  |
|  | ) |  |  |  |

Remove a specified pair in a specified structure.

Break the pair between i and i's pairing partner Return 0 if there is no error. Return 3 if structurenumber out of range. Return 4 if nucleotide number out of range.

**Parameters:**
:   |  |  |  |
    | --- | --- | --- |
    |  | *i* | is the index of a nucleotide in a pair that will be broken. |
    |  | *structurenumber* | is an integer specifying the structure from which to remove the pairs. |

**Returns:**
:   An integer that indicates an error code that can be parsed by GetErrorMessage() or GetErrorMessageString(), 0 = no error.

|  |  |  |  |  |
| --- | --- | --- | --- | --- |
| void RNA::RemoveConstraints | ( |  | ) |  |

Remove all folding constraints.

This function strips all previously assigned folding constraints. Note that this function does not delete SHAPE constraints or pseudo free energies.

|  |  |  |  |  |  |
| --- | --- | --- | --- | --- | --- |
| int RNA::RemovePairs | ( | const int | *structurenumber* = `1` | ) |  |

Remove all the current base pairs in a specified structure.

Return 0 if there is no error. Return 5 if structurenumber never had pairs specified.

**Parameters:**
:   |  |  |  |
    | --- | --- | --- |
    |  | *structurenumber* | is an integer specifying the structure from which to remove the pairs. |

**Returns:**
:   An integer that indicates an error code that can be parsed by GetErrorMessage() or GetErrorMessageString(), 0 = no error.

|  |  |  |  |  |
| --- | --- | --- | --- | --- |
| int RNA::SetExtrinsic | ( | int | *i*, |  |
|  |  | int | *j*, |  |
|  |  | double | *k* |  |
|  | ) |  |  |  |

Add extrinsic restraints for partition function calculations.

This function multiplies the equilibrium constant for structures including the i-j basepair by k. This applies only to partition functions and to stochastic traceback. If k>1, then the i-j pair is favored and if k<1, the i-j pair is disfavored. k should always be >= 0. In case of error, the function returns a non-zero that can be parsed by GetErrorMessage() or GetErrorMessageString().

**Parameters:**
:   |  |  |  |
    | --- | --- | --- |
    |  | *i* | is the index of a nucleotide in the i-j pair. |
    |  | *j* | is the index of the other nucleotide in the i-j pair. |
    |  | *k* | is an equilibrium constant that is >= 0. |

**Returns:**
:   An integer that indicates an error code (0 = no error, >0 indicates an error).

|  |  |  |  |  |  |
| --- | --- | --- | --- | --- | --- |
| void RNA::SetModifier | ( | std::string | *modifier* | ) |  |

Sets the chemical modifier type for the data contained in the SHAPE constraints file. Can be SHAPE, DMS, or CMCT.

**Parameters:**
:   |  |  |  |
    | --- | --- | --- |
    |  | *modifier* | is either SHAPE, DMS, or CMCT, depending on the chemical modifier. |

|  |  |  |  |  |  |
| --- | --- | --- | --- | --- | --- |
| void RNA::SetProgress | ( | TProgressDialog & | *Progress* | ) |  |

Provide a TProgressDialog for following calculation progress. A TProgressDialog class has a public function void update(int percent) that indicates the progress of a long calculation.

**Parameters:**
:   |  |  |  |
    | --- | --- | --- |
    |  | *Progress* | is a TProgressDialog class. |

Reimplemented in HybridRNA.

|  |  |  |  |  |
| --- | --- | --- | --- | --- |
| int RNA::SpecifyPair | ( | const int | *i*, |  |
|  |  | const int | *j*, |  |
|  |  | const int | *structurenumber* = `1` |  |
|  | ) |  |  |  |

Specify a base pair between nucleotides i and j.

The base pair is in structure number structurenumber, which is assumed to be structure 1. Return 0 if there is no problem, otherwise return an error code: error = 3 -> structurenumber out of range. error = 4 -> nucleotide number out of range. A c string or string description of the error are available using GetErrorMessage() or GetErrorMessageString(). Note!: Sequences with the 5' end = nucleotide 1. Note!: Structures start at structure 1.

**Parameters:**
:   |  |  |  |
    | --- | --- | --- |
    |  | *i* | is an integer for the position of the first nucleotide in the pair. |
    |  | *j* | in an integer for the position of the second nucleotide in the pair. |
    |  | *structurenumber* | is the structure that has the pair. This defaults to 1. |

**Returns:**
:   An integer that indicates an error code that can be parsed by GetErrorMessage() or GetErrorMessageString(), 0 = no error.

|  |  |  |  |  |
| --- | --- | --- | --- | --- |
| int RNA::Stochastic | ( | const int | *structures*, |  |
|  |  | const int | *seed* = `1` |  |
|  | ) |  |  |  |

Sample structures from the Boltzman ensemable.

This function requires partition function data from either a previous partition function calculations or from having read a partition function save file during construction of the class. In case of error, the function returns a non-zero that can be parsed by GetErrorMessage() or GetErrorMessageString().

**Parameters:**
:   |  |  |  |
    | --- | --- | --- |
    |  | *structures* | is the number of structures to be sampled. |
    |  | *seed* | is an integer that seeds the random number generator that is required for sampling, which defaults to 1. |

**Returns:**
:   An int that indicates an error code (0 = no error, non-zero = error).

|  |  |  |  |  |
| --- | --- | --- | --- | --- |
| void RNA::StopProgress | ( |  | ) |  |

Provide a means to stop using a TProgressDialog. StopProgress tells the RNA class to no longer follow progress. This should be called if the TProgressDialog is deleted, so that this class does not make reference to it.

Reimplemented in HybridRNA.

|  |  |  |  |  |  |
| --- | --- | --- | --- | --- | --- |
| int RNA::WriteConstraints | ( | const char | *filename*[] | ) |  |

Write the current set of folding constraints to disk in a plain text file.

This function does not write SHAPE pseudo energies.

**Parameters:**
:   |  |  |  |
    | --- | --- | --- |
    |  | *filename* | is a c string that is the file name to be written. |

**Returns:**
:   An integer that indicates an error code (0 = no error). Currently, this function does not generate errors, but the return is provided to add error handling in the future.

|  |  |  |  |  |
| --- | --- | --- | --- | --- |
| int RNA::WriteCt | ( | const char | *filename*[], |  |
|  |  | bool | *append* = `false` |  |
|  | ) |  |  |  |

Write a ct file of the structures.

Return 0 if no error and non-zero errors can be parsed by GetErrorMessage() or GetErrorMessageString().

**Parameters:**
:   |  |  |  |
    | --- | --- | --- |
    |  | *filename* | is a NULL terminated c string that specifies the name of the ct file to be written. |
    |  | *append* | is a bool that indiactes whether the ct data should be appended to an existing file. If true, data will be appended if the file exists, or a new file created if the file does not exist. If false, any esiting file is overwritten. This is false by default. |

**Returns:**
:   An integer that provides an error code. 0 = no error, 10 = no structure to write.

|  |  |  |  |  |  |
| --- | --- | --- | --- | --- | --- |
| int RNA::WriteDotBracket | ( | const char | *filename*[] | ) |  |

Write dot-bracket file of structures.

Return 0 if no error and non-zero errors can be parsed by GetErrorMessage() or GetErrorMessageString().

**Parameters:**
:   |  |  |  |
    | --- | --- | --- |
    |  | *filename* | is a NULL terminated c string that specified the name of the file to be written. |

**Returns:**
:   An integer that provides an error code. 0 = no error.

|  |  |  |  |  |
| --- | --- | --- | --- | --- |
| int RNA::WriteThermodynamicDetails | ( | const char | *filename*[], |  |
|  |  | const bool | *UseSimpleMBLoopRules* = `false` |  |
|  | ) |  |  |  |

Calculate the folding free energy change for all structures and write the details of the calculation to a file.

Free energies are in kcal/mol. The first time this is called, if no other free energy calculation has been performed and the folding temperature has not been specifed, thermodynamic parameter files (.dat) files will be read from disk. The parameter files should be located in the directory specified by environment variable $DATAPATH, or the pwd. In case of error, the function returns a non-zero.

**Parameters:**
:   |  |  |  |
    | --- | --- | --- |
    |  | *filename* | is a NULL terminated c string that provides the name of the output file to be written. |
    |  | *UseSimpleMBLoopRules* | is a bool that indicates what energy rules to use. The default, false, uses the complete nearest neighbor model for multibranch loops. When true is passed, the energy model is instead a simplified model that is the one used by the dynamic programming algorithms. |

**Returns:**
:   An int that indicates whether an error occurred (0 = no error; 5 = error reading parameter files).

---

## Member Data Documentation

structure\* RNA::ct `[private]`

bool RNA::drawallocated `[private]`

bool RNA::energyallocated `[private]`

int RNA::ErrorCode `[protected]`

arrayclass\* RNA::ev `[private]`

arrayclass \* RNA::ew `[private]`

arrayclass\* RNA::ew2 `[private]`

integersize \* RNA::ew3 `[private]`

integersize\* RNA::ew5 `[private]`

arrayclass \* RNA::ewmb `[private]`

arrayclass \* RNA::ewmb2 `[private]`

forceclass\* RNA::fce `[private]`

bool\* RNA::lfce `[private]`

bool \* RNA::mod `[private]`

bool RNA::partitionfunctionallocated `[private]`

pfdatatable\* RNA::pfdata `[private]`

TProgressDialog\* RNA::progress `[protected]`

PFPRECISION RNA::Q `[private]`

coordinates\* RNA::structurecoordinates `[private]`

pfunctionclass \* RNA::v `[private]`

int RNA::vmin `[private]`

pfunctionclass\* RNA::w `[private]`

PFPRECISION \* RNA::w3 `[private]`

PFPRECISION\* RNA::w5 `[private]`

PFPRECISION \*\* RNA::wca `[private]`

pfunctionclass \* RNA::wcoax `[private]`

pfunctionclass \* RNA::wl `[private]`

pfunctionclass \* RNA::wmb `[private]`

pfunctionclass \* RNA::wmbl `[private]`

---

The documentation for this class was generated from the following files:

- F:/RNAstructure-co-from-repository/RNAstructure/RNA\_class/RNA.h- F:/RNAstructure-co-from-repository/RNAstructure/RNA\_class/RNA.cpp

---

Generated on Tue Jul 30 10:52:29 2013 for RNAstructure Classes by 
 1.5.7.1
